# *Arid1b* haploinsufficiency in parvalbumin- or somatostatin-expressing interneurons leads to distinct ASD-like and ID-like behavior

**DOI:** 10.1038/s41598-020-64066-5

**Published:** 2020-05-12

**Authors:** Amanda L. Smith, Eui-Man Jung, Byeong Tak Jeon, Woo-Yang Kim

**Affiliations:** 1 Department of Pharmacology and Experimental Neuroscience, University of Nebraska Medical Center, Omaha, NE 68198 USA; 2 Laboratory of Veterinary Biochemistry and Molecular Biology, College of Veterinary Medicine, Chungbuk National University, Cheongju, 28644 Republic of Korea; 3 Department of Biological Sciences, Kent State University, Kent, OH 44242 USA

**Keywords:** Developmental biology, Neuroscience

## Abstract

Inhibitory interneurons are essential for proper brain development and function. Dysfunction of interneurons is implicated in several neurodevelopmental disorders, including autism spectrum disorder (ASD) and intellectual disability (ID). We have previously shown that *Arid1b* haploinsufficiency interferes with interneuron development and leads to social, cognitive, and emotional impairments consistent with ASD and ID. It is unclear, however, whether interneurons play a major role for the behavioral deficits in *Arid1b* haploinsufficiency. Furthermore, it is critical to determine which interneuron subtypes contribute to distinct behavioral phenotypes. In the present study, we generated *Arid1b* haploinsufficient mice in which a copy of the *Arid1b* gene is deleted in either parvalbumin (PV) or somatostatin (SST) interneurons, and examined their ASD- and ID-like behaviors. We found that *Arid1b* haploinsufficiency in PV or SST interneurons resulted in distinct features that do not overlap with one another. *Arid1b* haploinsufficiency in PV neurons contributed to social and emotional impairments, while the gene deletion in the SST population caused stereotypies as well as learning and memory dysfunction. These findings demonstrate a critical role of interneurons in *Arid1b* haploinsufficient pathology and suggest that PV and SST interneurons may have distinct roles in modulating neurological phenotypes in *Arid1b* haploinsufficiency-induced ASD and ID.

## Introduction

Autism spectrum disorder (ASD) and intellectual disability (ID) are highly prevalent neurodevelopmental disorders, characterized by social communication impairments and cognitive dysfunction, respectively^1,2^. Emotional disturbance such as aggressive behavior, depression, and anxiety is another significant aspect of ASD and ID^3–8^. While behaviors of ASD and ID are relatively well characterized, the neuropathogenesis of these conditions is poorly understood. Accordingly, no pharmacologic or genetic interventions are available for ASD or ID. Haploinsufficiency of the *AT-rich interactive domain 1B* (*ARID1B*) gene has been shown to cause ASD and ID in humans^9–11^. ARID1B is a component of the Brg/Brm associated factor (BAF) chromatin remodeling complex in the brain^12,13^. It binds to DNA through its AT-rich DNA-binding domain and alters chromatin structure, facilitating transcription factor access and regulating gene expression.

Many neurodevelopmental disorders exhibit improper inhibitory interneuron development, resulting in excitatory/inhibitory (E/I) imbalance^14–16^. We have previously generated an *Arid1b* mouse model and showed that *Arid1b* haploinsufficient (*Arid1b*^+/−^) mice recapitulate ASD and ID behavior^17^. Importantly, *Arid1b* haploinsufficient mice exhibit a reduction and abnormal distribution of interneurons as well as abnormal inhibitory synaptic activity in the cerebral cortex^17^. No clear anatomical or physiological phenotype has been found in excitatory neurons, suggesting a more prominent effect on interneuron abnormalities in potentially creating a range of social, intellectual, and emotional deficits in *Arid1b* haploinsufficient mice.

Thus, we hypothesized that *Arid1b* haploinsufficiency-induced neurological behavior is mediated by interneurons. To test this idea, we conditionally knocked out the *Arid1b* gene in parvalbumin (PV) and somatostatin (SST) interneurons using specific Cre-drivers and examined neurological behaviors in interneuron-specific *Arid1b* haploinsufficient mice. PV and SST neurons are the most populous interneuron subtypes in the cortex, each constituting 30–40% of the inhibitory interneuron population^18^. These subtypes exhibit a range of different morphological, electrophysiological, and molecular properties, and appear to play roles in distinct circuit functions^19,20^. Little is known regarding how these individual interneuron subtypes modulate various behaviors. Here, we show that *Arid1b* haploinsufficiency in PV and SST neurons has distinct behavioral phenotypes that recapitulate behavioral deficits seen in *Arid1b* haploinsufficient mice. PV interneurons with *Arid1b* haploinsufficiency alter social and emotional behaviors, while SST interneurons of the gene mutation affect learning and stereotyped behaviors. Our findings demonstrate that interneurons mediate *Arid1b* haploinsufficiency-induced behaviors and suggest distinct roles of PV and SST interneurons for ASD and ID behavior.

## Results

### *Arid1b* haploinsufficiency in PV and SST interneurons have distinct effects on social and stereotyped behaviors

**Figure 1:**
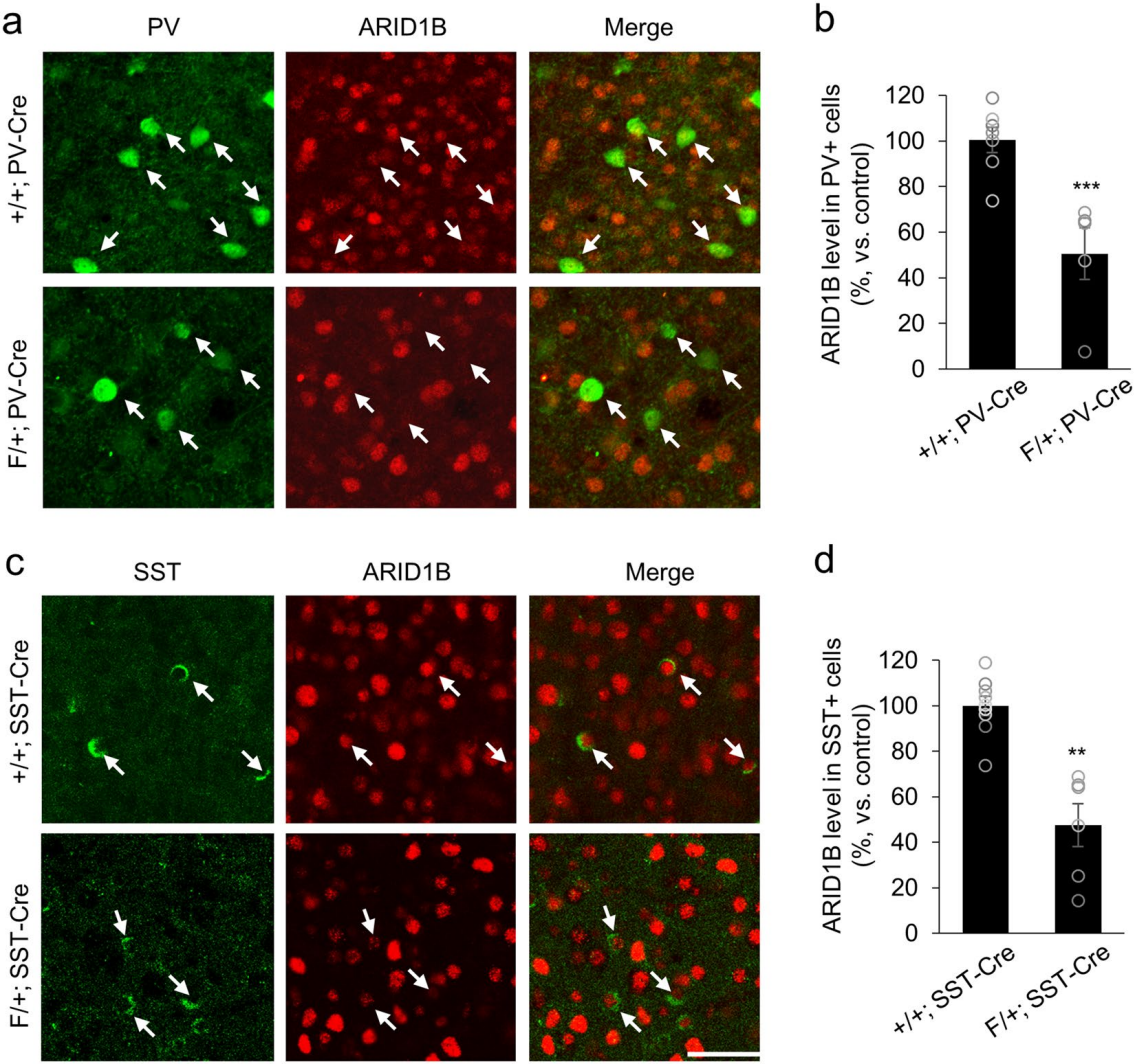
ARID1B expression in PV and SST interneurons of *Arid1b* mutant mice. (**a**) Cortical sections from P60 +/+; PV-Cre and F/+; PV-Cre mice were co-immunolabeled with antibodies to ARID1B and PV. (**b**) Quantification of ARID1B levels in PV interneurons of +/+; PV-Cre and F/+; PV-Cre mice (***p < 0.0001; n = 4 for +/+; PV-Cre mice and n = 5 for F/+; PV-Cre mice; two-tailed Student’s t test). (**c**) Cortical sections from P60 +/+; SST-Cre and F/+; SST-Cre mice were co-immunolabeled with ARID1B and SST. (**d**) Quantification of ARID1B levels in SST interneurons of +/+; SST-Cre and F/+; SST-Cre mice (**p = 0.0017; n = 3 for +/+; SST-Cre mice and n = 4 for F/+; SST-Cre mice; two-tailed Student’s t test). Data shown are mean ± SEM.

Our previous study has shown that *Arid1b* haploinsufficient (*Arid1b*^+/−^) mice exhibit social, emotional, and cognitive deficits as seen in ASD and ID^17^. To determine the contributions of PV and SST interneurons to the ASD- and ID-like behaviors observed in *Arid1b*^+/−^ mice, we generated two lines of conditional knockout mice lacking one copy of *Arid1b* in either PV (F/+; PV-Cre) or SST (F/+; SST-Cre) neurons by crossing floxed *Arid1b* mice with PV-Cre or SST-Cre mice, respectively. F/+; PV-Cre and F/+; SST-Cre mice show no changes in overall body or brain weight compared to littermate controls (Figs. S1 and S2). To verify successful generation of conditional knockout mice, we examined ARID1B expression by co-labeling cortical sections with antibodies to ARID1B and PV or ARID1B and SST (Fig. 1). The level of ARID1B was decreased in the F/+; PV-Cre cortex by 42% compared to the protein level in control samples (Fig. 1a,b). A reduction in the ARID1B level by 48% was also observed in F/+; SST-Cre tissues (Fig. 1c,d). Next, we assessed inhibitory synapses in F/+; PV-Cre and F/+; SST-Cre mice by immunolabelling inhibitory synaptic markers, vesicular inhibitory amino acid transporter (VIAAT) and glutamic acid decarboxylase2 (GAD2). We found that the numbers of inhibitory synaptic puncta positive to VIAAT and GAD2 were decreased in F/+; PV-Cre and F/+; SST-Cre mice compared to controls (Figs. S3 and S4). The reduced formation of inhibitory synapses suggests that interneuron properties are disrupted in these mutant mice. Conversely, there was no difference in the number of excitatory synaptic puncta positive to vesicular glutamate transporter 1 (VGLUT1) between control and mutant mice (Figs. S1 and S2). Western blotting confirmed the morphological changes, revealing the decreased level of VIAAT in mutant brain lysates (Figs. S3 and S4). Full length Western blots are presented in Supplementary Fig. S6.

**Figure 2:**
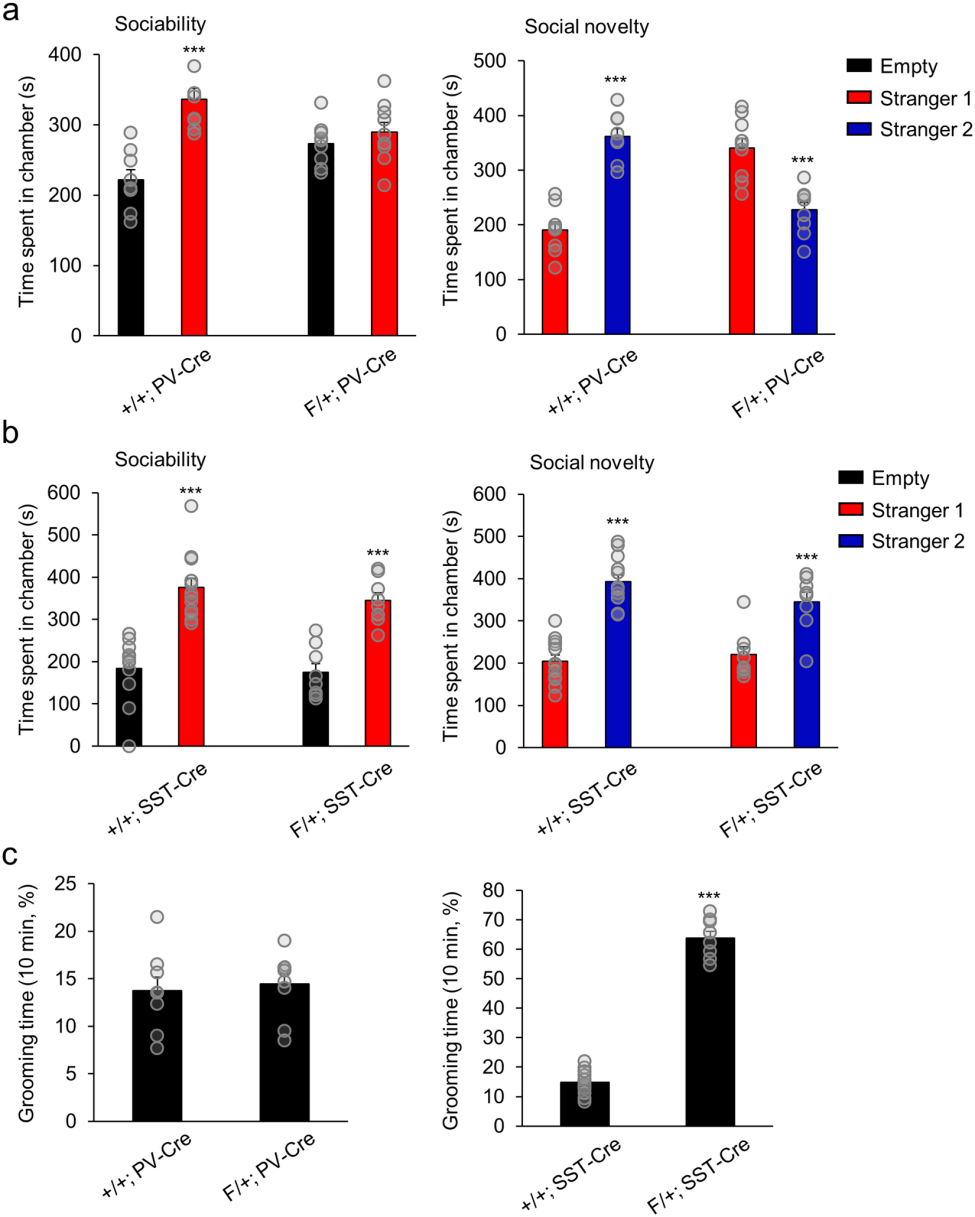
Social and stereotyped behaviors in F/+; PV-Cre and F/+; SST-Cre mice. (**a**) In the three-chamber test for sociability, F/+; PV-Cre mice spent a similar amount of time with the empty cage as with the stranger mouse (stranger 1), whereas control counterparts stayed more time with stranger 1 (+/+; PV-Cre: ***p = 0.0002; F/+; PV-Cre: p = 0.3701; n = 8 mice for +/+; PV-Cre and n = 9 mice for F/+; PV-Cre; one-way ANOVA followed by Bonferroni post hoc test). In the three-chamber test for social novelty, F/+; PV-Cre mice spent less time with the novel stranger (stranger 2) than with the familiar mouse (stranger 1), in contrast to controls (+/+; PV-Cre: ***p < 0.0001; F/+; PV-Cre: ***p = 0.0002; n = 8 mice for +/+; PV-Cre and n = 9 mice for F/+; PV-Cre; one-way ANOVA followed by Bonferroni post hoc test). (**b**) F/+; SST-Cre mice showed increased time spent with stranger 1 compared with the empty cage in the sociability test, similar to their control counterparts (+/+; SST-Cre: ***p < 0.0001; F/+; SST-Cre: ***p < 0.0001; n = 12 mice for +/+; SST-Cre and n = 8 mice for F/+; SST-Cre; one-way ANOVA followed by Bonferroni post hoc test). F/+; SST-Cre also spent more time with the novel stranger compared to stranger 1 during the social novelty test, which is comparable to control mice (+/+; SST-Cre: ***p < 0.0001; F/+; SST-Cre: ***p = 0.0012; n = 12 mice for +/+; SST-Cre and n = 8 mice for F/+; SST-Cre; one-way ANOVA followed by Bonferroni post hoc test). (**c**) F/+; PV-Cre mice exhibited a similar level of grooming time compared to +/+; PV-Cre mice (p = 0.7022; n = 8 mice for +/+; PV-Cre and n = 9 mice for F/+; PV-Cre; two-tailed Student’s t test). F/+; SST-Cre mice, however, showed increased grooming time compared to +/+; SST-Cre mice (***p < 0.0001; n = 12 mice for +/+; SST-Cre and n = 8 mice for F/+; SST-Cre; two-tailed Student’s t test). Data shown are mean ± SEM.

We characterized these mice with multiple behavioral assays. *Arid1b* wild-type littermates with a Cre driver (+/+; PV-Cre or +/+; SST-Cre) were used as controls. We first examined social and stereotyped behaviors. Social impairments and repetitive behaviors are two core features of ASD. We assessed sociability and social novelty using the three-chamber test. In the sociability test, +/+; PV-Cre control mice spent more time in the chamber containing the stranger 1 mouse than in the empty chamber, whereas F/+; PV-Cre mice showed no preference for either chamber. In the social novelty test, control mice showed preference for the novel stranger 2 mouse, whereas F/+; PV-Cre mice spent more time in the chamber containing the more familiar stranger 1 mouse (Fig. 2a). In contrast, F/+; SST-Cre mice showed a preference for the chamber with the unfamiliar stranger mouse in both the sociability and social novelty tests, similar to their control counterparts (Fig. 2b). We then examined the incidence of repetitive, stereotyped behaviors by looking at spontaneous grooming. F/+; PV-Cre mice showed no change in the amount of time spent self-grooming compared to +/+; PV-Cre controls (Fig. 2c). On the other hand, F/+; SST-Cre mice exhibited an increase in self-grooming time in comparison to control mice (Fig. 2c). Together, these results show that *Arid1b* haploinsufficiency in PV neurons produces deficits in social behaviors, while the haploinsufficiency in SST neurons leads to excessive repetitive behaviors.

### *Arid1b* haploinsufficiency in PV interneurons, but not SST interneurons, results in heightened anxiety and depression-like phenotype

**Figure 3:**
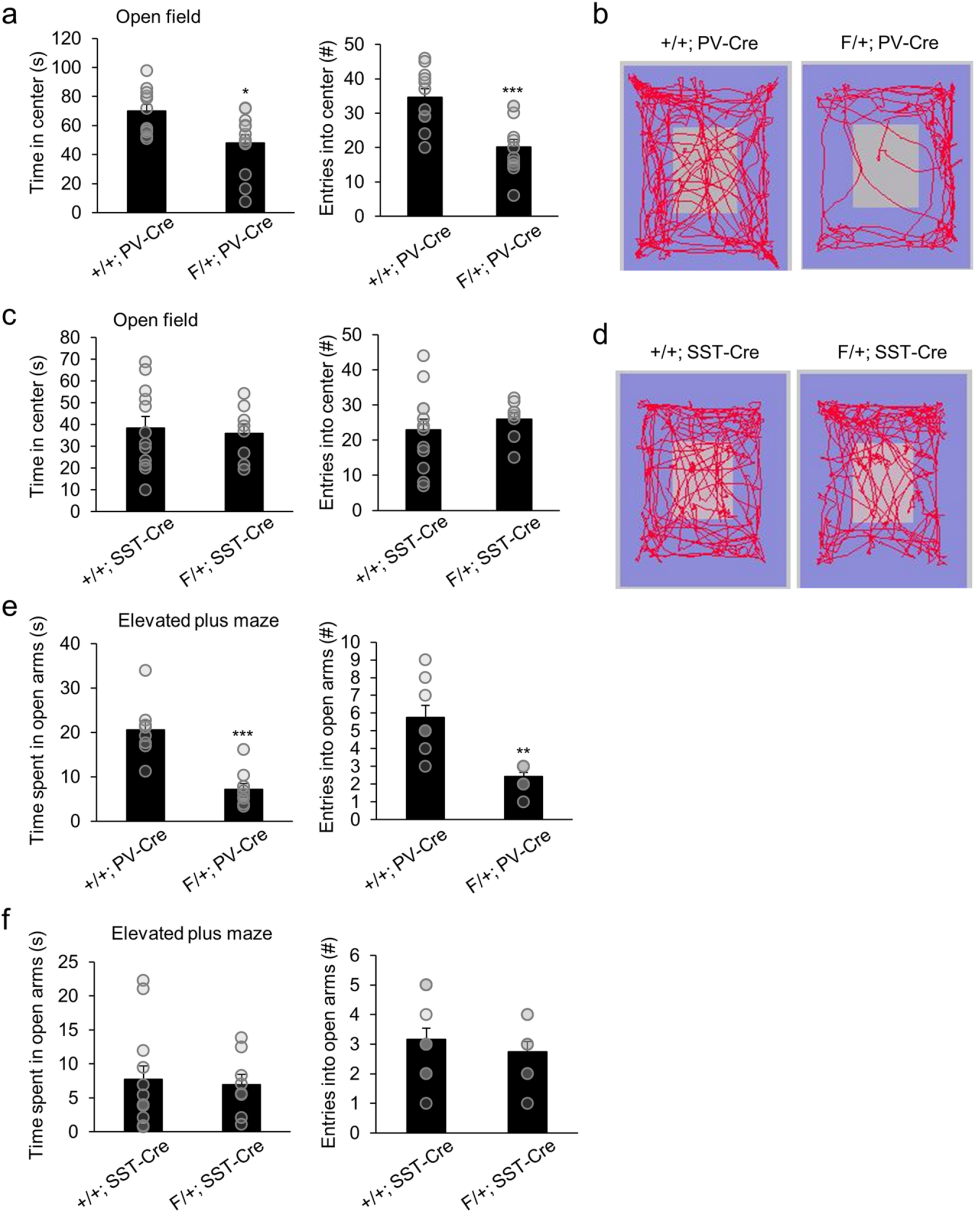
Heightened anxiety in F/+; PV-Cre mice. (**a**) In the open field test, time spent in the center and number of entries into the center were both decreased in F/+; PV-Cre mice compared to +/+; PV-Cre mice (time in center: *p = 0.0100; center entries: ***p = 0.0004; n = 11 mice for +/+; PV-Cre and n = 12 mice for F/+; PV-Cre; two-tailed Student’s t test). (**b**) Representative tracings of the open field activity in +/+; PV-Cre and F/+; PV-Cre mice. (**c**) F/+; SST-Cre mice showed a similar amount of time spent in the center and similar number of entries into the center compared to +/+; SST-Cre mice in the open field test (time in center: p = 0.7632; center entries: p = 0.4300; n = 12 mice for +/+; SST-Cre and n = 8 mice for F/+; SST-Cre; two-tailed Student’s t test). (**d)** Representative tracings of the open field activity in +/+; SST-Cre and F/+; SST-Cre mice. (**e**) In the elevated plus maze, F/+; PV-Cre mice exhibited less time in open arms and fewer entries into open arms compared to control counterparts (time in open arms: ***p = 0.0001; open arm entries: **p = 0.0019; n = 8 mice for +/+; PV-Cre and n = 9 mice for F/+; PV-Cre; two-tailed Student’s t test). (**f**) F/+; SST-Cre mice showed no change in time spent in open arms or total entries into open arms compared to +/+; SST-Cre mice (time in open arms: p = 0.8053; open arm entries: p = 0.4670; n = 12 mice for +/+; SST-Cre and n = 8 mice for F/+; SST-Cre; two-tailed Student’s t test). Data shown are mean ± SEM.

Because heightened levels of anxiety and depression are among the most common comorbid disorders in those with ASD, we next investigated the contribution of interneuron subtypes in these psychiatric conditions in *Arid1b* conditional knockout mice. The open field test was performed to gauge the levels of anxiety in F/+; PV-Cre and F/+; SST-Cre mice. We found that F/+; PV-Cre mice made fewer entries and spent less time in the center of the open field compared to +/+; PV-Cre controls, preferring to stay on the edges and in corners of the arena (Fig. 3a,b). However, F/+; SST-Cre mice showed no differences in anxiety behavior compared to control mice, spending similar amounts of time in the center of the open field (Fig. 3c,d). Both F/+; PV-Cre and F/+; SST-Cre mice exhibited no changes in the total distance traveled or average velocity compared to wild type mice, confirming no confounding locomotor deficits (Fig. S5a,b). We crosschecked the emotional change in F/+; PV-Cre mice with the elevated plus maze test. F/+; PV-Cre mice exhibited greater levels of anxiety compared to controls, making fewer entries and spending less time in the open arms (Fig. 3e). F/+; PV-Cre mice also spent more time in the closed arms compared to controls (Fig. S5c). There were no differences in the amount of time spent and the number of entries into the open arms (Fig. 3f) or time spent in closed arms (Fig. S5d) between controls and F/+; SST-Cre mice. Again, these results suggest PV interneuron dysfunction as a mediator of *Arid1b* haploinsufficiency-induced anxiety phenotypes.

**Figure 4:**
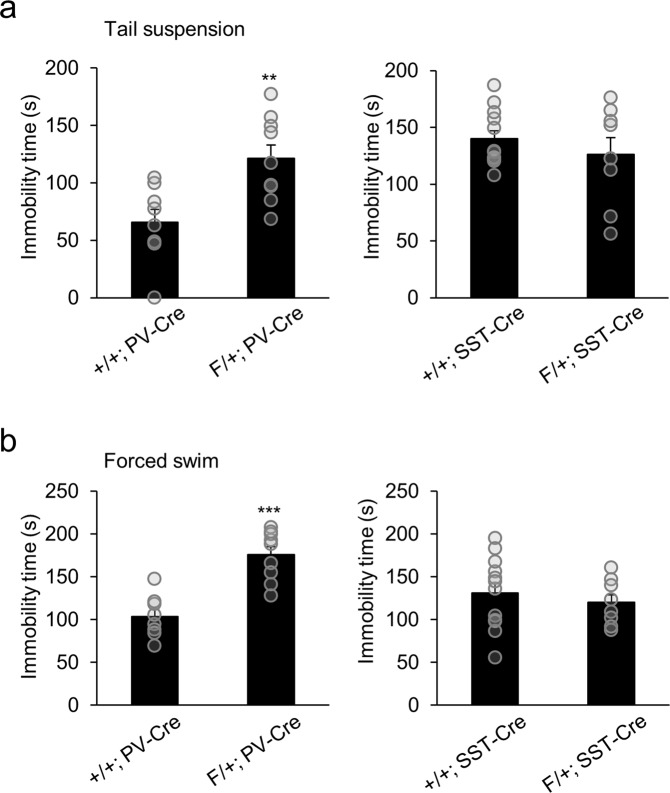
Increased depression behavior in F/+; PV-Cre mice. (**a**) In the tail suspension test, F/+; PV-Cre mice showed increased immobility time compared to control mice (**p = 0.0055; n = 8 mice for +/+; PV-Cre and n = 9 mice for F/+; PV-Cre; two-tailed Student’s t test). F/+; SST-Cre mice showed no change in immobility time compared to controls in the tail suspension test (p = 0.4381; n = 12 mice for +/+; SST-Cre and n = 8 mice for F/+; SST-Cre; two-tailed Student’s t test). (**b**) In the forced swim test, F/+; PV-Cre mice showed increased immobility time compared to controls (***p < 0.0001; n = 8 mice for +/+; PV-Cre and n = 9 mice for F/+; PV-Cre; two-tailed Student’s t test). F/+; SST-Cre mice showed no change in immobility time in the forced swim test compared to their controls (p = 0.5225; n = 12 mice for +/+; SST-Cre and n = 8 mice for F/+; SST-Cre; two-tailed Student’s t test). Data shown are mean ± SEM.

The level of depression-like behavior was also measured by the tail suspension and forced swim tests. F/+; PV-Cre mice displayed heightened levels of depression-like behavior, indicated by an increase in total immobility time in the tail suspension test compared to control mice (Fig. 4a). However, there was no significant difference in immobility time between F/+; SST-Cre and control mice, suggesting little change in depression-like behavior. The forced swim test of F/+; PV-Cre and F/+; SST-Cre mice resulted in a similar pattern to the tail suspension test. F/+; PV-Cre mice revealed an increased time of immobility compared to control mice while F/+; SST-Cre mice showed no significant change (Fig. 4b). These results demonstrate that PV-specific *Arid1b* haploinsufficiency leads to similar anxiety and depression-like phenotypes that are seen in global *Arid1b* haploinsufficiency. In contrast, SST-specific *Arid1b* haploinsufficiency appears to have little contribution to the emotional disturbance.

### Learning and memory deficits are caused by *Arid1b* haploinsufficiency in SST neurons

**Figure 5:**
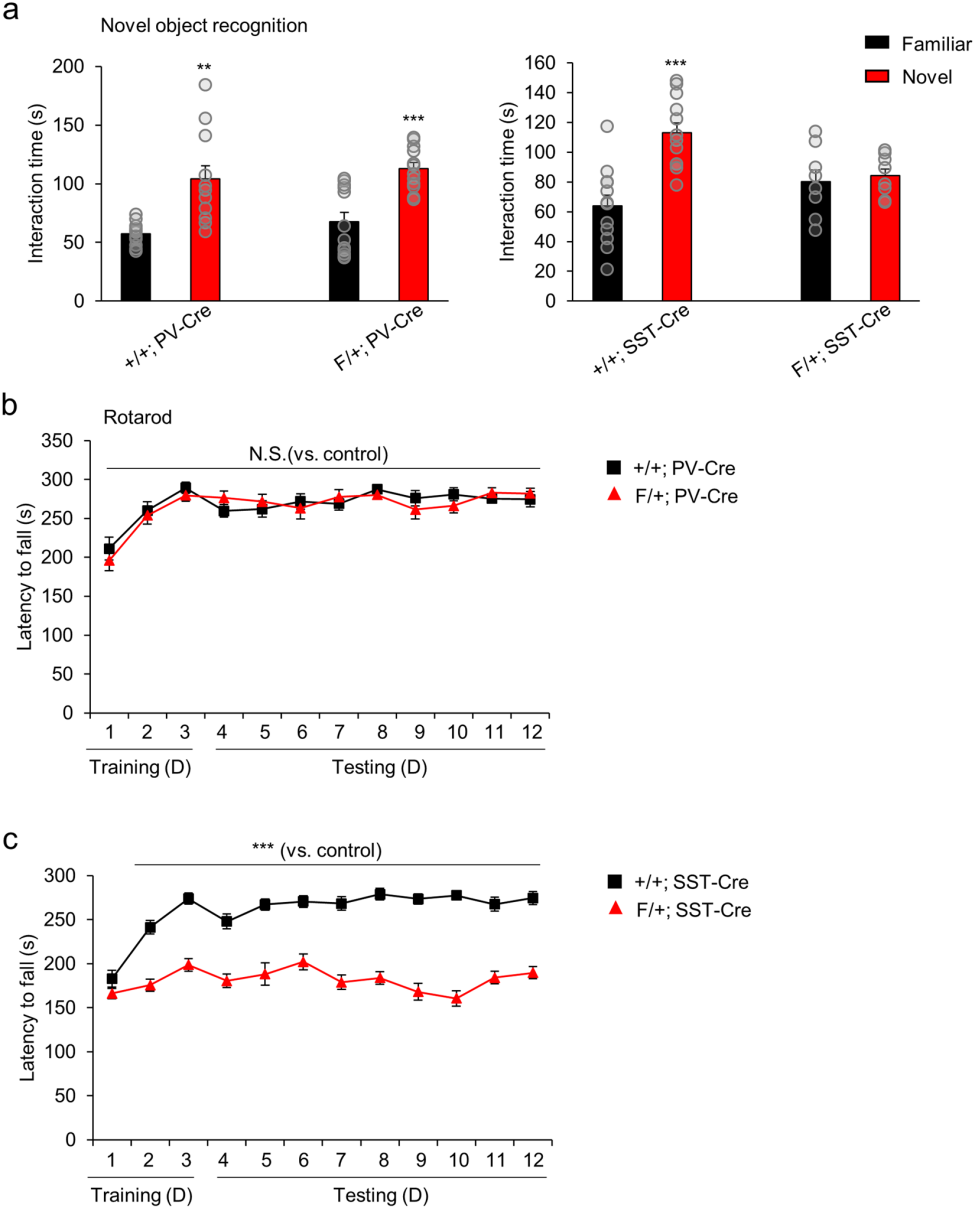
Recognition memory and motor learning deficits in F/+; SST-Cre mice. (**a**) In the novel object recognition test, both +/+; PV-Cre and F/+; PV-Cre mice showed greater interaction time with the novel object compared to the familiar object (+/+; PV-Cre: **p = 0.0032; F/+; PV-Cre: ***p = 0.0001; n = 11 mice for +/+; PV-Cre and n = 12 mice for F/+; PV-Cre; one-way ANOVA followed by Bonferroni post hoc test). F/+; SST-Cre mice showed no change in the amount of time spent with the novel object versus the familiar object, whereas +/+; SST-Cre mice interacted more with the novel object (+/+; SST-Cre: ***p < 0.0001; F/+; SST-Cre: p = 0.7009; n = 12 mice for +/+; SST-Cre and n = 8 mice for F/+; SST-Cre; one-way ANOVA followed by Bonferroni post hoc test). (**b**) +/+; PV-Cre and F/+; PV-Cre mice exhibited similar latencies to fall during both the training and testing phases of the rotarod test (d1: p = 0.4500; d2: p = 0.6978; d3: p = 0.3718; d4: p = 0.2056; d5: p = 0.5397; d6: p = 0.6487; d7: p = 0.5175; d8: p = 0.4032; d9: p = 0.4076; d10: p = 0.2905; d11: p = 0.4571; d12: p = 0.5813; n = 8 mice for +/+; PV-Cre and n = 8 mice for F/+; PV-Cre; two-way ANOVA followed by Bonferroni post hoc test). (**c**) F/+; SST-Cre mice showed a decreased latency to fall during the rotarod test from day 2 to day 12 (d1: p = 0.1556; d2: ***p < 0.0001; d3: ***p < 0.0001; d4: ***p < 0.0001; d5: ***p = 0.0004; d6: ***p < 0.0001; d7: ***p < 0.0001; d8: ***p < 0.0001; d9: ***p < 0.0001; d10: ***p < 0.0001; d11: ***p < 0.0001; d12: ***p < 0.0001; n = 8 mice for +/+; SST-Cre and n = 8 mice for F/+; SST-Cre; two-way ANOVA followed by Bonferroni post hoc test). Data shown are mean ± SEM.

Severe cognitive impairments, the primary feature of ID, are found in *Arid1b*^+/−^ mice^17^. To examine learning and memory functions in F/+; PV-Cre and F/+; SST-Cre mice, we used several behavior assays of testing different types of learning and memory. We first performed the novel object recognition test to assess recognition memory. Control mice spent more time with a novel object than with a familiar object. F/+; PV-Cre mice behaved similar to controls, approaching and interacting more with the novel object (Fig. 5a). In contrast, F/+; SST-Cre mice showed no preference for exploring either the familiar or novel object, suggesting impairments in recognition memory. In the rotarod test for motor learning, both F/+; PV-Cre mice and controls showed a strong increase in the latency to fall during the three-day training phase (Fig. 5b). Throughout the testing phase (days 4 to 12), both control and F/+; PV-Cre mice revealed a consistent latency to fall. However, F/+; SST-Cre mice did not show the same increase in the latency to fall compared to controls during the training phase (Fig. 5c). In addition, they showed decreased latencies to fall throughout the testing phase compared to control mice, suggesting deficits in motor learning ability.

**Figure 6:**
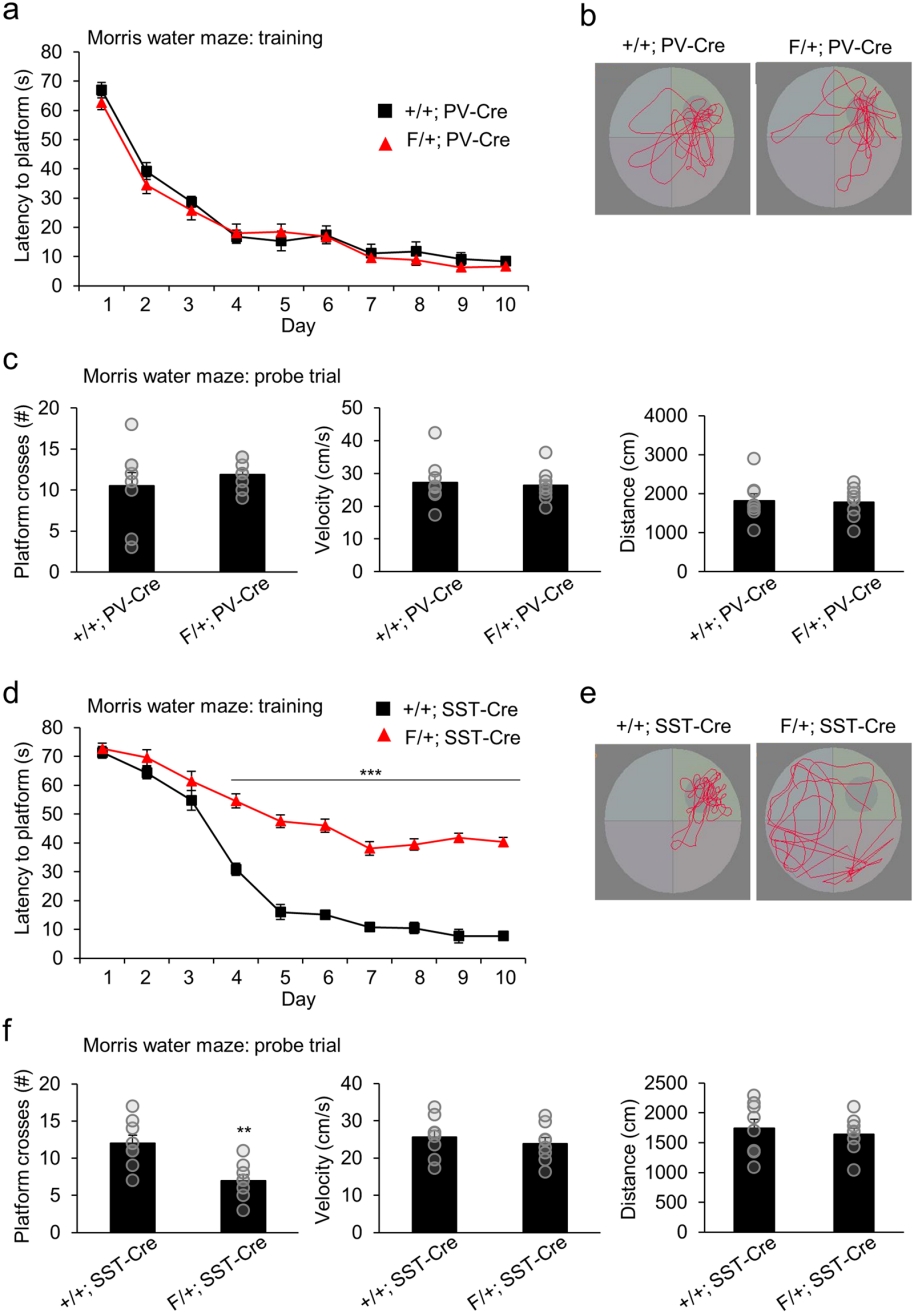
Dysfunctional spatial learning and memory in F/+; SST-Cre mice. (**a**) The training phase of the Morris water maze test showed no change in latency to the platform in F/+; PV-Cre mice compared to control mice (d1: p = 0.2811; d2: p = 0.2898; d3: p = 0.4904; d4: p = 0.7700; d5: p = 0.5078; d6: p = 0.8765; d7: p = 0.6736; d8: p = 0.4795; d9: p = 0.2874; d10: p = 0.3262; n = 8 mice for +/+; PV-Cre and n = 8 mice for F/+; PV-Cre; two-way ANOVA followed by Bonferroni post hoc test). (**b**) Representative swim paths of +/+; PV-Cre and F/+; PV-Cre mice during a probe trial after training. (**c)** During a probe trial, F/+; PV-Cre mice showed no change in the number of platform crosses, average velocity, or total distance traveled compared to controls (platform crosses: p = 0.4776; average velocity: p = 0.7694; distance traveled: p = 0.8522; n = 8 mice for +/+; PV-Cre and n = 8 mice for F/+; PV-Cre; two-tailed Student’s t test). (**d)** F/+; SST-Cre mice showed an increased latency to the platform from day 4 to day 10 during the training phase of the Morris water maze test (d1: p = 0.6765; d2: p = 0.1794; d3: p = 0.2084; d4: ***p < 0.0001; d5: ***p < 0.0001; d6: ***p < 0.0001; d7: ***p < 0.0001; d8: ***p < 0.0001; d9: ***p < 0.0001; d10: ***p < 0.0001; n = 8 mice for +/+; SST-Cre and n = 8 mice for F/+; SST-Cre; two-way ANOVA followed by Bonferroni post hoc test). (**e)** Representative swim paths of +/+; SST-Cre and F/+; SST-Cre mice during a probe trial after training. (**f)** During a probe trial, F/+; SST-Cre mice showed fewer platform crosses compared to control mice, while average velocity and total distance traveled were unchanged (platform crosses: **p = 0.0037; average velocity: p = 0.5127; distance traveled: p = 0.5982; n = 8 mice for +/+; SST-Cre and n = 8 mice for F/+; SST-Cre; two-tailed Student’s t test). Data shown are mean ± SEM.

To test spatial learning and reference memory, we next performed the Morris water maze test. During the 10-day training phase, F/+; PV-Cre mice showed similar latencies to the hidden platform as control mice (Fig. 6a). In a probe trial to assess spatial memory following training, F/+; PV-Cre mice crossed the platform location a similar number of times as controls (Fig. 6b,c). These mice also showed no difference in the total swimming distance or speed (Fig. 6c). F/+; SST-Cre mice showed similar latencies to the platform compared to controls for the first three days of training, but then exhibited higher latency times throughout the remainder of the training phase (Fig. 6d). Thus, F/+; SST-Cre mice may have an ability to initiate the learning process, but the capacity of further spatial learning appears to be limited. They crossed the platform location fewer times compared to controls during the probe trial (Fig. 6e,f). No difference was observed in the total swimming distance or speed, verifying that these factors did not contribute to the decrease in platform crosses (Fig. 6f). Together, these results suggest that SST-specific *Arid1b* haploinsufficiency leads to several learning and memory impairments.

## Discussion

*Arid1b* haploinsufficiency causes ASD and ID, leading to a broad range of neurobehavioral phenotypes in humans and rodents^9–11,17,21,22^. Given the abnormal anatomy and physiology of interneurons as a major hallmark of *Arid1b* haploinsufficiency^17^, we hypothesized that interneurons play a critical role in mediating *Arid1b* haploinsufficiency-induced behavioral impairments. The heterogenous nature of the interneuron population led us to investigate the contributions of the two most populous interneuron subtypes, PV and SST, to the behavioral phenotypes associated with *Arid1b* haploinsufficiency. Our results suggest that PV and SST interneurons contribute distinct, complementary aspects of the major ASD- and ID-like phenotypes in *Arid1b*^+/−^ mice. F/+; PV-Cre and F/+; SST-Cre mice recapitulate the major deficits induced by *Arid1b* haploinsufficiency (Table 1).

**Table 1:** Summary of behavioral deficits.

Behavior	*Arid1b*^+/−^	F/+; PV-Cre	F/+; SST-Cre
Social behavior	↓	↓	—
Stereotyped behavior	↑	—	↑
Anxiety	↑	↑	—
Depression-like behavior	↑	↑	—
Recognition memory	↓	—	↓
Motor learning	↓	—	↓
Spatial reference memory	↓	—	↓

Perturbation of the well-balanced excitatory and inhibitory components can lead to life-long cognitive, social, and emotional disabilities. Impairments in inhibitory interneuron development and function have been implicated in several neurodevelopmental conditions including ASD and ID^23–28^, Rett syndrome^29,30^, Schizophrenia^31^, Angelman syndrome^32^, and fragile X syndrome^33–35^. Particularly, it has been increasingly evident that PV or SST interneurons are dysfunctional in ASD and ID. Several studies have shown altered PV interneuron number and function in rodent models of ASD and ASD-related disorders^33,36–39^. Furthermore, post-mortem brains of patients with ASD and ID show fewer PV interneurons within the cerebral cortex^40,41^. Fewer SST neurons within the cerebral cortex has also been found in a *Pten* knockout mouse model of ASD^42^. It is likely that the anatomical or functional impairments of these interneurons cause the excitatory/inhibitory imbalance, resulting in abnormal neurobehaviors.

Although inhibitory interneurons are implicated in neurodevelopmental pathology, it has been unclear whether their subtypes play unique roles in distinct neural behavior. In this study using *Arid1b* mouse models, we show that PV interneurons are strongly associated with social behavior deficits, a core feature of ASD. These results are consistent with previous studies showing a prominent role of PV neurons in social behavior. *Pvalb* knockout mice display social behavior deficits and reduced vocalizations^43^. Reducing inhibitory output by knocking out an NMDA receptor specifically in PV neurons also results in reduced sociability in mice^44^. In addition, we show an important role of PV interneurons in emotional behavior. Recent studies support this finding as manipulation of PV interneurons modulates anxiety and depression-like behavior^45–47^. Our results show no clear association between SST interneurons and emotional behavior. However, previous studies have reported that SST interneurons are involved in anxiety and depression-like behavior^48–51^. In post-mortem examination of humans with major depressive disorder, there is reduced expression of SST in the prefrontal cortex^52,53^. There are some possibilities for the contradicting results. First, a loss or reduction of SST might have a more dramatic effect on SST neurons, whereas haploinsufficiency of the *Arid1b* gene in SST cells could result in less potency on the interneuron activity. It is likely that *Arid1b* haploinsufficiency elicits a different downstream mechanism than removing the SST peptide. Also, expression of the *Arid1b* gene could be different in interneuron subtypes and brain regions. In areas strongly associated with emotional behavior, such as the amygdala and pre-frontal cortex, *Arid1b* may be expressed at a lower level compared to other regions.

We further show that learning and memory dysfunction in the *Arid1b* haploinsufficient condition is modulated by SST interneurons. Studies have revealed the prominent role of SST interneurons in various cognitive functions. The SST subtype controls disinhibition of principle cells in the hippocampus, regulating sensory processing, learning, and memory^54^. This subtype also inhibits distal dendrites of principal neurons in layer 1 of the motor cortex to regulate changes in excitatory synapses during motor learning^21^. SST neurons in the prefrontal cortex maintains working memory during a delay phase^55^. Studies have also shown that signaling downstream of the SST peptide in interneurons affects learning and memory. For example, knockout of the *Type 3 somatostatin receptor* (*Sstr3*) or *Type 2 somatostatin receptor* (*Sstr2*) gene inhibits SST signaling, which results in impaired object recognition memory^56^ or spatial learning and reference memory^57^, respectively. These findings suggest a significant contribution of SST interneurons to learning and memory behaviors.

There is no treatment tool available for the core symptoms of ASD and ID. Current pharmacologic strategies focus on GABAergic modulation to normalize inhibitory activity. GABA agonists to enhance inhibition, such as benzodiazepines and anticonvulsants, have been helpful in alleviating aggressive and hyperactive behaviors associated with ASD in humans^58,59^. Use of clonazepam, a positive modulator of the GABA_A_ receptor, to enhance inhibitory activity has also successfully rescued behavioral phenotypes in mouse models of ASD and ID^17,60^. A prominent shortcoming of these strategies is that they are currently not subtype-specific to inhibitory neurons. As we continue to gain a better understanding of how individual interneuron subtypes modulate distinct behaviors in ASD and/or ID, therapeutic strategies could become even more targeted, with greater efficacy and fewer side effects as a result.

## Materials and Methods

### Western blotting

Proteins (40 μg/lane) were separated on 4-15% SDS-PAGE gel and transferred to immobilon-P membrane (Millipore) by a Trans-Blot SD semi-dry transfer cell (Bio-Rad Laboratories). Following primary antibodies were used: anti-VIAAT (PhosphoSolutions, rabbit, 2100-VGAT, 1:1000) and anti-GAPDH (Millipore, mouse, MAB374, 1:10,000). GAPDH was used as an internal control to normalize band intensity. Density measurements were performed by using NIH ImageJ. Background samples from an area near each lane were subtracted from each band to acquire mean band density.

### *Arid1b* conditional knockout mice

The *Arid1b*-floxed allele^17^ was crossed with either the PV-Cre (B6;129P2-Pvalb^tm1(cre)Arbr^/J; JAX 008069) or SST-Cre (B6N.Cg-Sst^tm2.1(cre)Zjh^/J; JAX 018973) allele. After weaning, all mice were group housed (no more than 5 mice per cage) as a mix of genotypes on a 12-hour light/dark cycle. All mice used as “stranger mice” in behavioral assays were housed separately from experimental mice. All husbandry and experimental procedures were approved by and in accordance with the recommendation of the Institutional Animal Care and Use Committee (IACUC) of Kent State University and the University of Nebraska Medical Center (protocols #: 10-077-09, 464 WK 18-08).

### Immunostaining

Immunostaining of brain sections was performed as described previously^61,62^. The following primary antibodies were used: anti-Parvalbumin (Millipore, Rabbit, AB1572; 1:200), anti-Arid1b (Abcam, Mouse, ab57461; 1:200), anti-VIAAT (PhosphoSolutions, Rabbit, 2100-VGAT, 1:500), anti-VGLUT1 (Millipore, Guinea pig, AB5905, 1:1000), GAD2 (Developmental Studies Hybridoma Bank, Rat, GAD6, 1:200), and anti-Somatostatin (MilliporeSigma, Rat, MAB354; 1:200). 4′,6-diamidino-2-phenylindole (DAPI; Sigma; 1μg/ml) was used for counterstaining. Appropriate secondary antibodies conjugated with Alexa Fluor dyes (Invitrogen) were used to detect primary antibodies. All slides were visualized and imaged under FV3000 (Olympus) fluorescent confocal microscope system. To quantify ARID1B expression levels, we measured the intensity of ARID1B fluorescence in PV or SST positive cells using NIH ImageJ. The expression intensities were averaged and presented after background subtraction and normalization to those of PV or SST negative cells.

### Three-chamber test

A rectangular, transparent Plexiglas box (60 × 40 cm, Ugobasile) divided by walls with openings into three equal-sized compartments was used. Before starting any behavior testing, test mice were habituated to the empty apparatus for 5 minutes. For sociability testing, the test mouse was placed in the center chamber (chamber 2), an empty wire enclosure was placed in chamber 1, and an unfamiliar stimulus mouse designated as “stranger 1” was placed in a wire enclosure in chamber 3. The test mouse was allowed to explore the entire apparatus for 10 minutes. For the social novelty test, the stranger 1 mouse in its wire enclosure was randomly placed in either chamber 1 or 3, and a novel mouse designated as “stranger 2” was taken from a different home cage and placed in the empty wire enclosure within the other flanking chamber. The test mouse was placed back into chamber 2 and allowed to explore the original stranger 1 or the novel stranger 2 for 10 minutes. Video recordings were taken of the test mouse during both sociability and social novelty tests. Time spent interacting with each wire enclosure was analyzed using video tracking EthoVision XT 7 software (Noldus). Both stranger mice were age- and sex-matched wild type mice.

### Grooming assessment

A test mouse was placed in a clear plastic cage (17 × 32 × 14 cm) with normal housing bedding. Food and water were removed. The mouse was habituated to the cage for 10 minutes, followed by a 10-minute testing period in which the mouse was allowed to explore the cage freely. The movement of the mouse was recorded by a camera during the testing period, and the total time spent grooming was analyzed. Head washing, body grooming, genital/tail grooming, and paw and leg licking were all considered grooming behavior.

### Open field test

A test mouse was placed near the wall of a 35 × 42 cm open field arena and allowed to explore freely for 5 minutes. The movement of the mouse during the 5-minute testing period was recorded by a camera. The number of entries into and the overall time spent in the center of the arena (15 ×15 cm imaginary square) were analyzed using EthoVision XT 7 software (Noldus). The open field arena was cleaned with ethanol and dried between each trial. The mice were not habituated to the arena before testing.

### Elevated plus maze test

The elevated plus maze was performed as previously described^17,63^. The apparatus (EB Instrument) was elevated 45 cm above the floor, and the test mouse was placed on the central platform (5 × 5 cm). The mouse was allowed to freely explore either the two open arms (35 × 5 cm) or two enclosed arms (35 × 5 × 15 cm) for 5 minutes. The number of entries into and total time spent in open and closed arms were recorded.

### Tail suspension test

A test mouse was suspended from 60 cm above the surface of a table using adhesive tape at the tip of the tail. The mouse was acclimatized for 1 minute, followed by a 5-minute testing period in which the total duration of immobility was measured. Passive, completely motionless hanging was considered immobile behavior.

### Forced swim test

A test mouse was placed into a plastic cylinder (20 cm height, 17 cm diameter) filled with room temperature water to a depth of 10 cm. The mouse was acclimatized for 1 minute, followed by a testing period of 5 minutes in which the duration of immobility was measured. A mouse floating motionlessly was considered immobile.

### Novel object recognition test

A test mouse was placed into an empty open field arena (35 × 42 cm) and allowed to explore freely during a 5-minute habituation period. The mouse was then removed and two objects of similar size (10.5 × 4.5 × 2.5 cm), but different shape and color, were placed in opposite corners of the arena, 7 cm from the side walls. The mouse was placed back into the arena and allowed to explore the two objects for 10 minutes. The mouse was returned to its home cage, and after 6 hours, one object was replaced with a novel object of a similar size but different shape and color than the previous object. The same test mouse was placed back in the arena to explore the familiar and novel objects for another 10 minutes while being recorded by a camera. The amount of time the mouse spent interacting with the two objects was analyzed using EthoVision XT software (Noldus).

### Rotarod test

Mice were placed on rotating drums (3 cm diameter) of an accelerating rotarod (Rotamex 4/8, Columbus Instruments International). Three 5-minute trials per day (at constant speeds of 4, 8, and 12 rpm) were performed for three days of training. The following nine testing days consisted of one test trial per day, with the speed of the rotarod accelerating from 4 to 40 rpm over a 5-minute period. The time taken for the mouse to fall off the rotating rod was measured for each trial and recorded as its latency to fall.

### Morris water maze test

A circular tank 110 cm in diameter and 91 cm in height (San Diego Instruments) was filled with water and divided into four equal quadrants (Q1-4) by lines drawn on the floor. Visual cues of different color and shape were present on the wall of each quadrant as a spatial reference. A 10 cm circular plexiglass platform was submerged 1 cm deep in Q2, hidden from the mice. For each trial, test mice were placed at the perimeter of the tank in one of four quadrants. Four trials were performed per mouse per day during a ten-day training phase. Each trial ended when the mouse climbed onto and remained on the hidden platform for ten seconds. The mouse was given 20 seconds to rest on the platform between trials. The time taken by the mouse to reach the platform was recorded as its latency. The time for four trials was averaged and recorded as a result for each mouse on each day. To test memory retention, the mice were subjected to a single 60-second probe trial on day 11. The hidden platform was removed and each mouse started the trial from Q4. The swim path was video recorded, and the number of annulus crossings, velocity, and swim distance were analyzed using EthoVision XT 7 tracking software (Noldus).

All behavior assays were performed during the light cycle. Health conditions, including weight, activity, and feeding were checked before assays. Male and female mice at ages 2–4 months were used for all behavior assays, and all assays were done in the same chronological order and timeframe. For social behavior assays, only male-male or female-female social interactions were examined to avoid interference of male-female sexual interactions and estrous cycle timing. All behavioral assays were done blind to genotypes, with age-matched littermates.

### Statistical analysis

Normal distribution was tested using the Kolmogorov-Smirnov test and variance was compared between populations. Statistical significance was determined using two-tailed, unpaired Student’s t-tests for two-population comparisons or one/two-way ANOVA followed by Bonferroni’s post hoc test for multiple comparisons. Data were analyzed using GraphPad Prism and presented as means ± SEM. *P* values and sample sizes for each comparison are described in figure legends.

## Supplementary information

Supplementary information.

## Data Availability

The datasets generated for the current study are available from the corresponding author upon request.
