# Supplementary material for: Arid1b haploinsufficiency in parvalbumin- or somatostatin-expressing interneurons leads to distinct ASD-like and ID-like behavior
Source: Sci Rep. 2020 May 12;10:7834. doi: 10.1038/s41598-020-64066-5 (PMC7217886; doi:10.1038/s41598-020-64066-5)

Title: ***Arid1b* haploinsufficiency in parvalbumin- or somatostatin-expressing interneurons leads to distinct ASD-like and ID-like behavior**

Amanda L Smith[1], Eui-Man Jung[2], Byeong-Tak Jeon[3], and Woo-Yang Kim[3*]

[1]Department of Pharmacology and Experimental Neuroscience, University of Nebraska Medical Center, Omaha, NE, 68198, USA

[2]Laboratory of Veterinary Biochemistry and Molecular Biology, College of Veterinary Medicine, Chungbuk National University, Cheongju, 28644, Republic of Korea

[3]Department of Biological Sciences, Kent State University, Kent, OH, 44242, USA

*Corresponding author

Correspondence to:

Woo-Yang Kim, Ph.D.

Email: wkim2@kent.edu

Phone: 330-672-7888

Supplementary information

Supplementary Figure 1. Body and brain weight with *Arid1b* haploinsufficiency in PV neurons.

a. Representative images of +/+; PV-Cre and F/+; PV-Cre mice. **b.** No change was found in overall body weight between +/+; PV-Cre and F/+; PV-Cre mice (p=0.435; n=4 mice for +/+; PV-Cre and n=5 mice for F/+; PV-Cre; two-tailed Student's t test). **c.** Representative images of isolated brains from +/+; PV-Cre and F/+; PV-Cre mice. **d.** There was no change in brain weight between +/+; PV-Cre and F/+; PV-Cre mice (p=0.909; n=4 mice for +/+; PV-Cre and n=5 mice for F/+; PV-Cre; two-tailed Student's t test). Data shown are mean ± SEM.

Supplementary Figure 2. Body and brain weight with *Arid1b* haploinsufficiency in SST neurons.

a. Representative images of +/+; SST-Cre and F/+; SST-Cre mice. **b.** Compared to +/+; SST-Cre mice, F/+; SST-Cre mice showed no difference in overall body weight (p=0.054; n=3 mice for +/+; SST-Cre and n=4 mice for F/+; SST-Cre; two-tailed Student's t test). **c.** Representative images of isolated brains from +/+; SST-Cre and F/+; SST-Cre mice. **d.** There was no change in brain weight between +/+; SST-Cre and F/+; SST-Cre mice (p=0.8075; n=3 mice for +/+; SST-Cre and n=4 mice for F/+; SST-Cre; two-tailed Student's t test). Data shown are mean ± SEM.

Supplementary Figure 3. Inhibitory and excitatory synapses in F/+; PV-Cre mice.

a. F/+; PV-Cre mice show altered inhibitory synapses in the cerebral cortex. Inhibitory and excitatory synapses in P60 +/+; PV-Cre and F/+; PV-Cre cortices were examined by immunostaining using antibodies to inhibitory presynaptic markers VIAAT and GAD2 and glutamatergic presynaptic marker VGLUT1. Scale bar = 10 μm. **b.** The numbers of inhibitory and excitatory synaptic puncta were quantified. **c.** Western blotting with an antibody to VIAAT was performed using cortical brain lysates from P60 +/+; PV-Cre and F/+; PV-Cre mice. **d.** Quantification of **c**. The VIAAT level was normalized to the GAPDH level. Data were shown as relative changes versus +/+; PV-Cre WT and described mean ± SEM. Statistical significance was determined by a two-tailed Student's *t* test.

Supplementary Figure 4. Inhibitory and excitatory synapses in F/+; SST-Cre mice.

a. Altered inhibitory synapses in the SST-Cre cortex. Inhibitory and excitatory synapses in P60 +/+; SST-Cre and F/+; SST-Cre cortices were examined. Scale bar = 10 μm. **b.** Quantification of synaptic numbers. **c.** Western blotting exhibited a decrease in the VIAAT level in the F/+; SST-Cre cortex. **d.** Quantification of **c**. The intensity was normalized to the GAPDH level. Data were shown as relative changes versus +/+; SST-Cre WT and described mean ± SEM. Statistical significance was determined by a two-tailed Student's *t* test.

Supplementary Figure 5. Travel distance and velocity in the open field test and closed arm time in the elevated plus maze test

a. In the open field test, F/+; PV-Cre mice exhibited no differences in the total distance traveled or average velocity compared to +/+; PV-Cre mice (distance traveled: p=0.8734;

average velocity: p=0.5062; n=11 mice for +/+; PV-Cre and n=12 mice for F/+; PV-Cre; two-tailed Student's t test). **b.** In the open field test, F/+; SST-Cre mice exhibited no differences in the total distance traveled or average velocity compared to +/+; SST-Cre mice (distance traveled: p=0.3861; average velocity: p=0.9968; n=12 mice for +/+; SST-Cre and n=8 mice for F/+; SST-Cre; two-tailed Student's t test). **c.** In the elevated plus maze, F/+; PV-Cre mice exhibited more time spend in closed arms compared to control counterparts (time in closed arms: ***p=0.0001; n=8 mice for +/+; PV-Cre and n=9 mice for F/+; PV-Cre; two-tailed Student's t test). **d.** In the elevated plus maze, F/+; SST-Cre mice spent a similar amount of time in closed arms compared to control counterparts (time in closed arms: p=0.6804; n=12 mice for +/+; SST-Cre and n=8 mice for F/+; SST-Cre; two-tailed Student's t test). Data shown are mean ± SEM.

Supplementary Figure 6. Inhibitory and excitatory synapses in F/+; SST-Cre mice.

Full length Western blots for cropped images in Fig. S3c and Fig. S4c.

Supplementary Figure 1

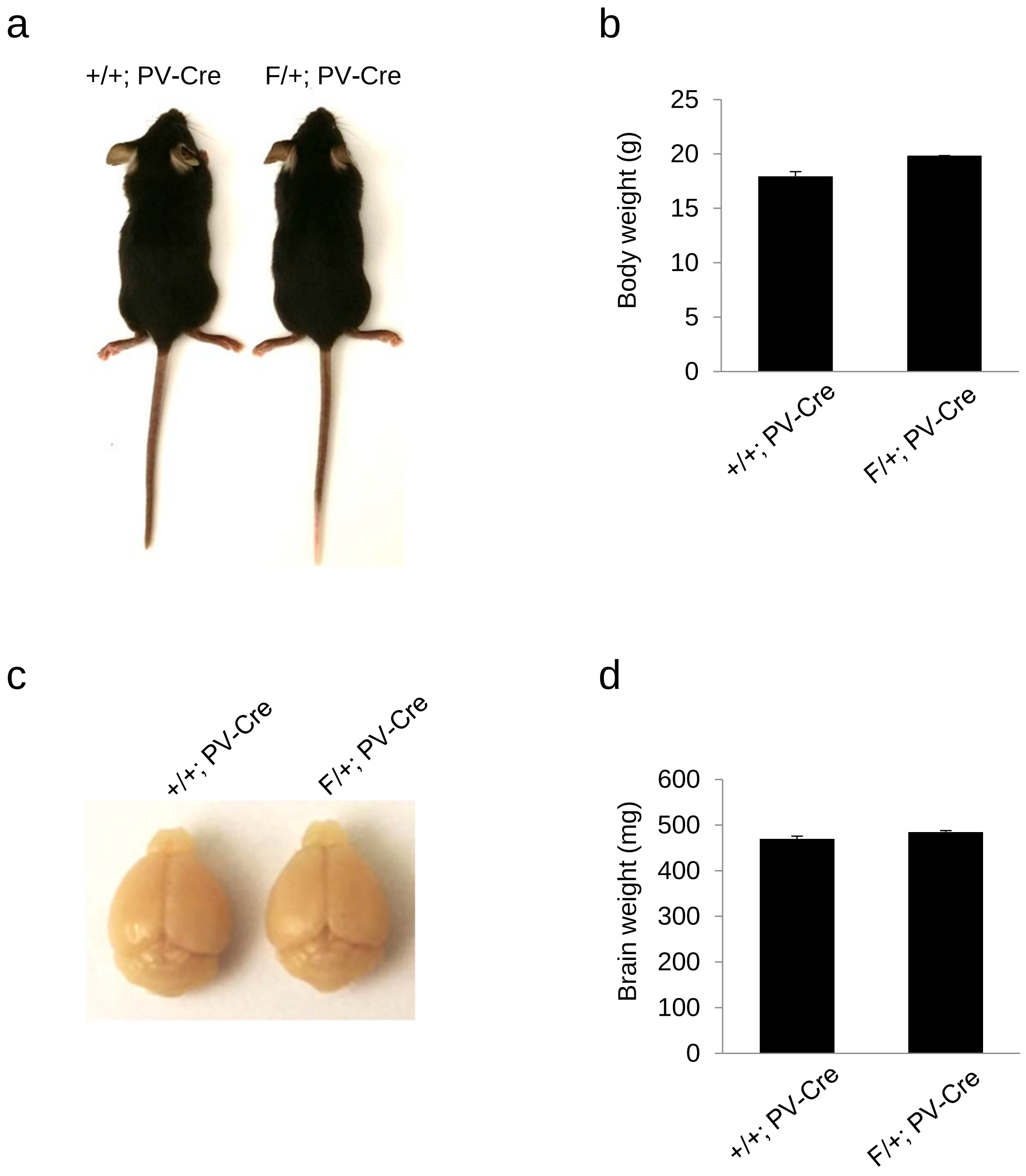

Supplementary Figure 2

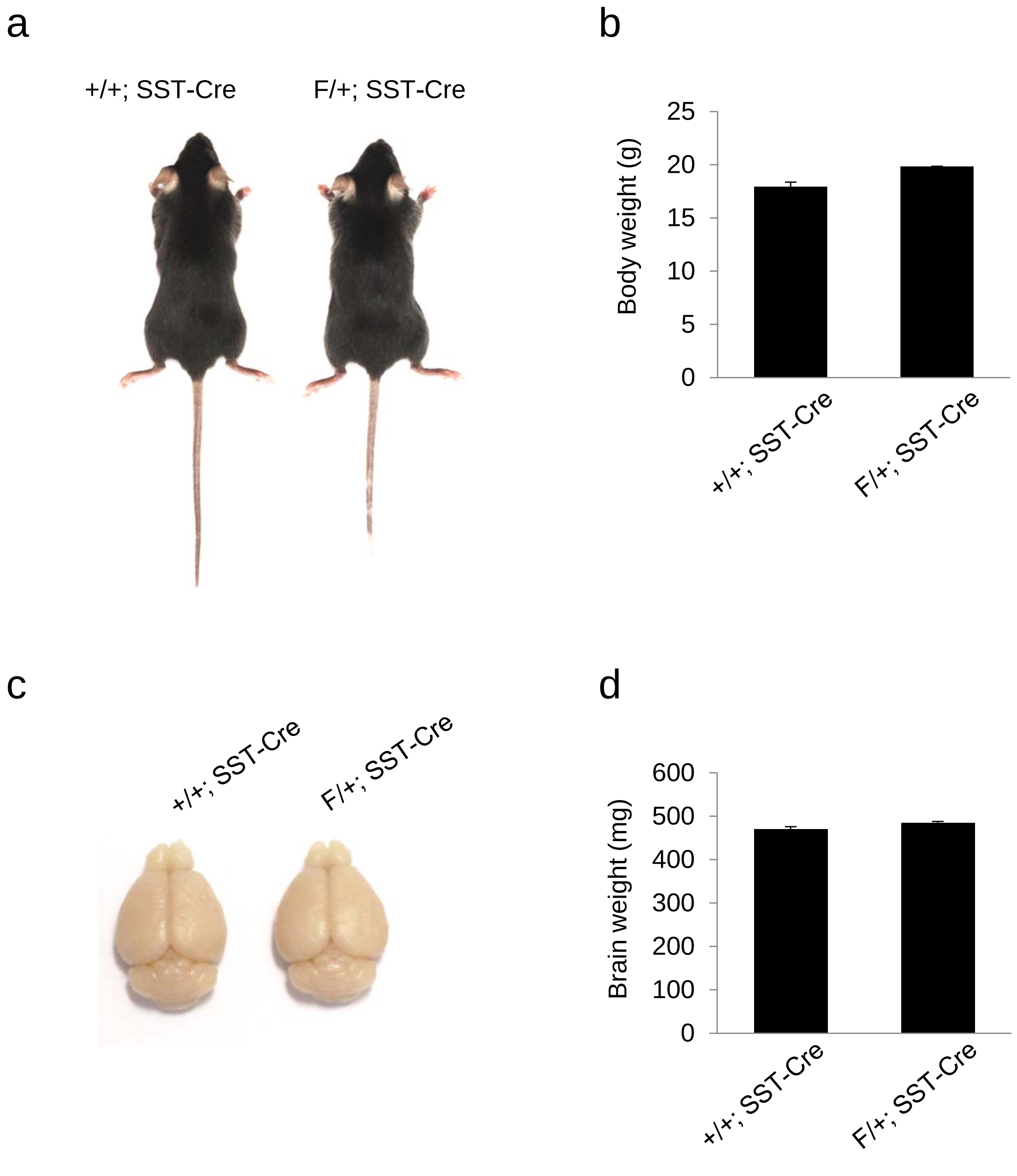

Supplementary Figure 3

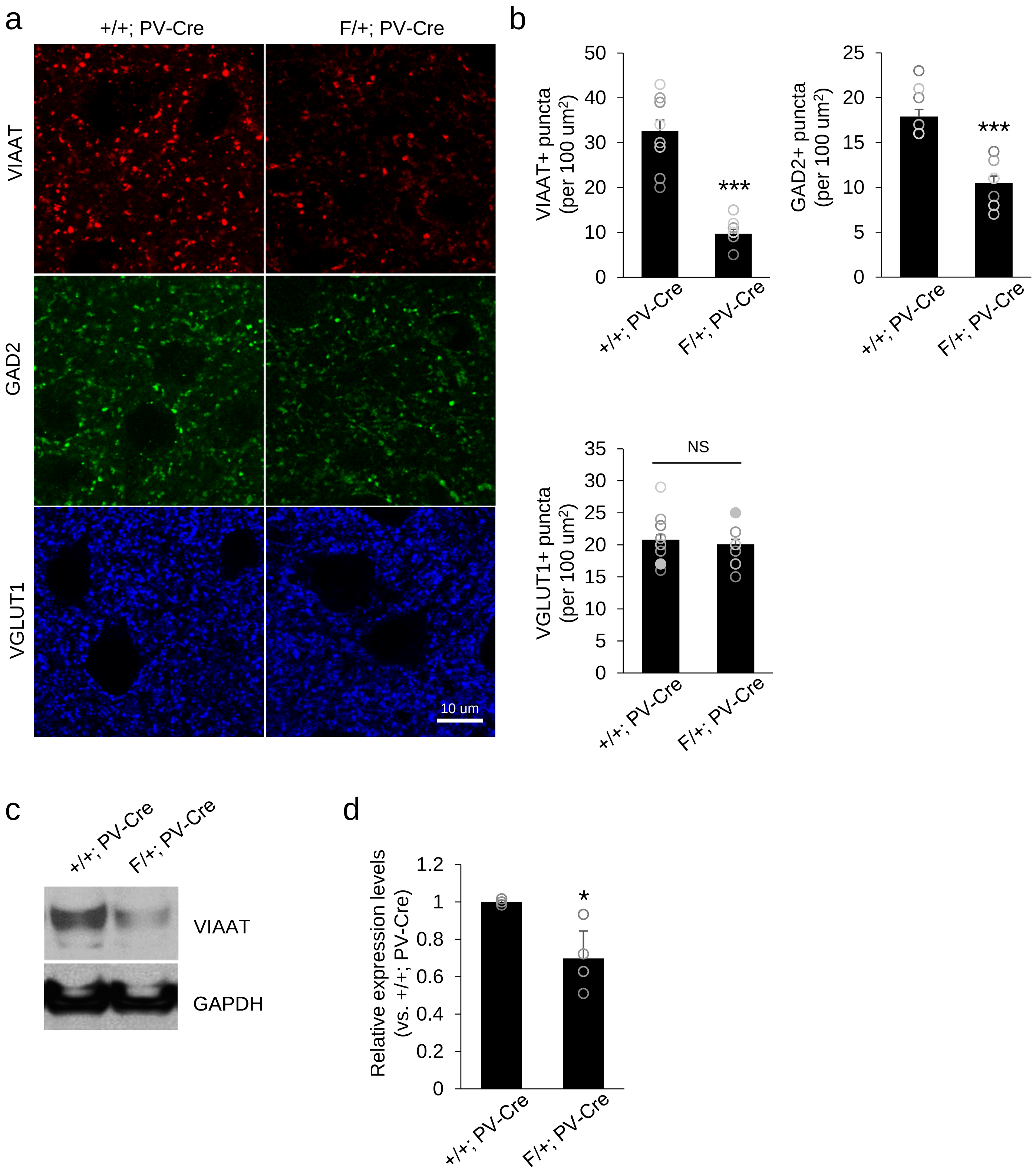

Supplementary Figure 4

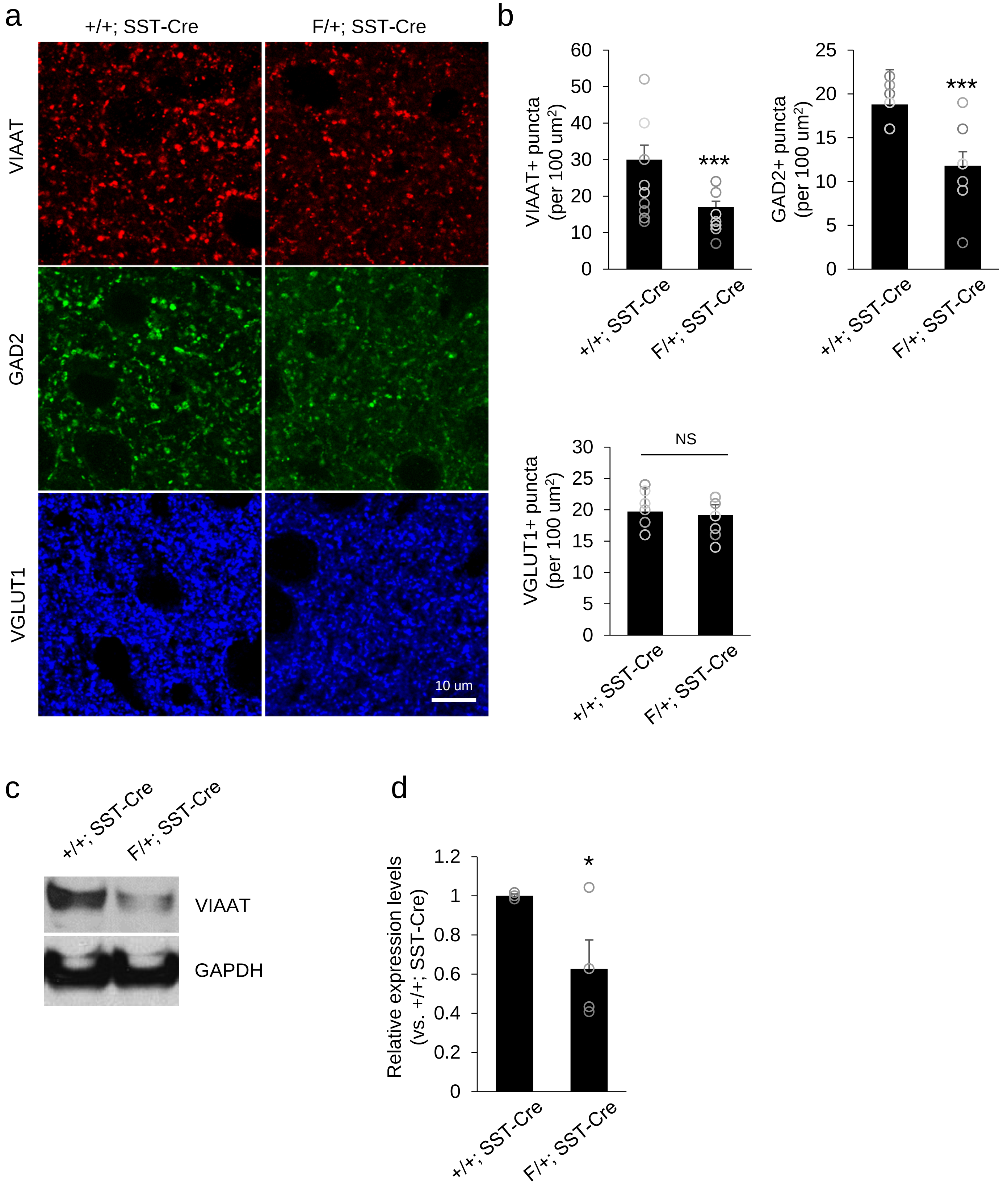

Supplementary Figure 5

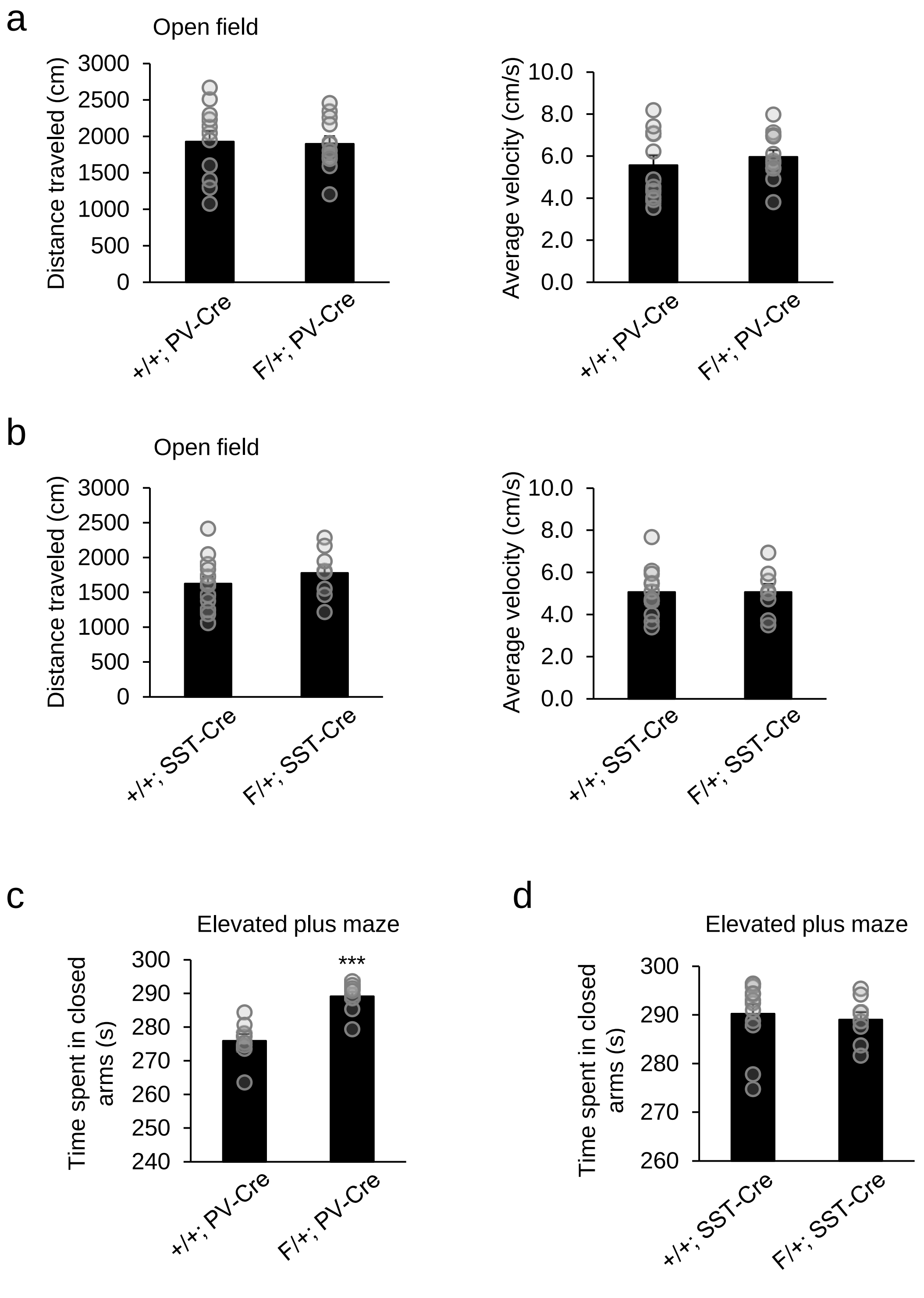

Supplementary Figure 6

Supplementary Fig. 3c

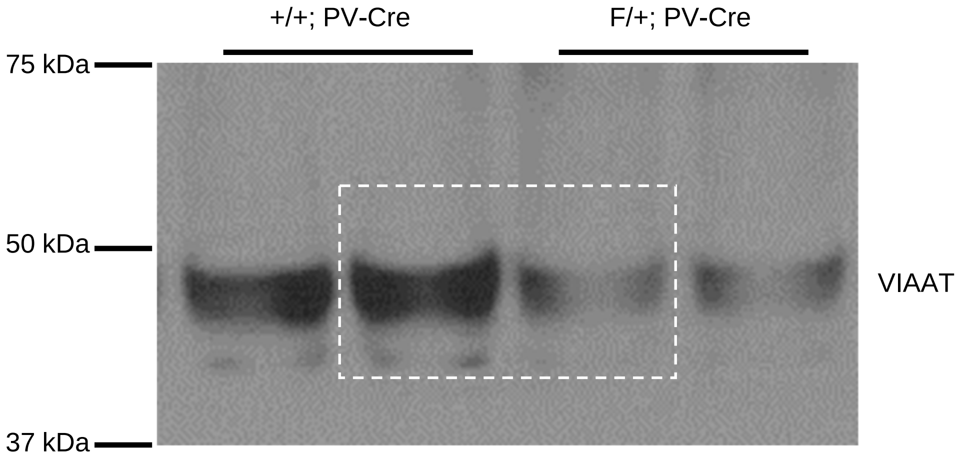

Supplementary Fig. 4c

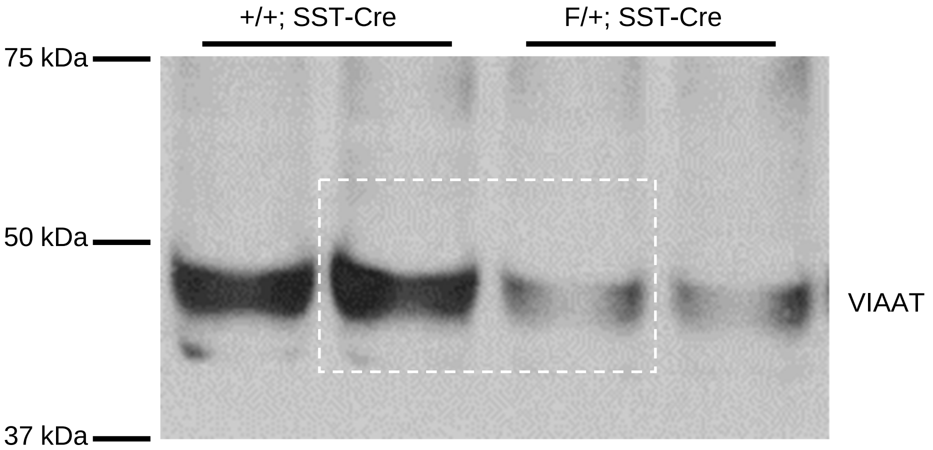

Supplement: Supplementary file 1 — Supplementary information. [file 41598_2020_64066_MOESM1_ESM.pdf]
